# Negative Correlation between miR-200c and Decorin Plays an Important Role in the Pathogenesis of Colorectal Carcinoma

**DOI:** 10.1155/2017/1038984

**Published:** 2017-04-16

**Authors:** Ren-Yi Tang, Zun Wang, Hong-Qi Chen, Si-Bo Zhu

**Affiliations:** ^1^Shanghai Starriver Bilingual School, 2588 Jindu Road, Minhang District, Shanghai 201108, China; ^2^Department of General Surgery, Affiliated Sixth People's Hospital, Shanghai Jiao Tong University, Shanghai, China; ^3^Shanghai Cinoasia Institute, Yangpu District, Shanghai 200437, China

## Abstract

*Aim.* To demonstrate the regulatory role of miRNA in colorectal carcinoma (CRC) and reveal the transcript markers that may be associated with CRC clinical outcomes.* Method.* Herein, we analyzed both mRNA and miRNA gene expression profiles of 255 CRC tumor samples from The Cancer Genome Atlas project to reveal the regulatory association between miRNA and mRNA. Also, the potential role of gene coexpression network in CRC has been explored.* Results.* The negative correlation between miR-200c and DCN (Decorin) was calculated in CRC, indicating that DCN could be a potential target of miR-200c. Clinical features indicated that colon polyp history and overall survival were significantly related to the expression level of miR-200c. Three coexpression networks have been constructed, and genes involved in the networks are related to cell cycle, NOTCH, and mTOR signaling pathways.* Conclusion*. Our result provides a new insight into cancer related mRNA coexpression network in CRC research.

## 1. Introduction

Colorectal carcinoma (CRC) is the third most commonly diagnosed and the fourth most deathly cancer, leading to 8% of all cancer deaths globally [1]. Among all complications, metastasis sharply reduces the survival rates of patients, with the five-year survival rate of 80 to 90 percent for patients with primary colon cancer compared to 5 to 10 percent for those with metastasis [2]. For years, researchers have tried to find biomarkers with high sensitivity and specificity for both efficient and early detection of cancer and proper prognostic treatment. For instance, carcinoembryonic antigen (CEA), prostate-specific antigen (PSA), carcinoma antigens (CA), circulating tumor DNA (ctDNA), and circulating tumor cells (CTCs) as biomarkers are now widely applied in the clinical practices [3]. Moreover, a new fluorescently labeled chimeric anti-CEA antibody was used for better detection and resection for colon cancer during operation [4]. However, more sensitive biomarkers are necessary in the accurate diagnostics and disease prognosis during biopsy practice.

Recently, miRNA, a group of noncoding, small RNA molecules, was found to be a powerful biomarker with bright prospect in cancer applications. MicroRNA participates in several pathophysiological processes and pathways, and its level is related to cancer burden and survival of cancer patients [5, 6]. Recent research also proved that miRNA correlated to cancer metastasis by modulating gene expression in signaling network [7]. For example, miR-146a is downregulated in esophageal cancer tissue and samples [8], while miR-221 is overexpressed in liver cancers [9] but downregulated in gastrointestinal stromal cancers [10]. Therefore, these small noncoding RNAs also serve as oncogenes or suppressors that depend on the type of miRNA [8]. miR-21 or miR-155, always working as oncogenes, is among the most common overexpressed miRNAs in human cancers [11]. In contrast, miR-15a/16-1 cluster, which is frequently deleted in chronic lymphocytic leukemia (CLL), works as cancer suppressor [12]. For colon cancer, report showed that the overexpression of miR-200c was correlated with metastatic behavior, so miR-200c could be considered as a potential biomarker for colorectal patients [13].

The Cancer Genome Atlas project is aimed at profiling genomic changes in more than 33 different cancer types including CRC [14]. TCGA applies high-throughput genomic sequencing techniques to enhance our understanding of the diagnosis, treatment, and prevention of cancer through a better understanding of the genetic basis of this disease. Herein, as to verify the mechanism of miRNA in colon cancer, we applied TCGA colon cancer dataset [14] for miRNA-mRNA association analysis. Patients' clinical manifestations were also related to miRNAs results. In addition, coexpression gene network analysis and pathway analysis were carried out to further analyze the roles of cancer related miRNAs.

## 2. Methods

### 2.1. Data Description and Preprocess

The sequencing based mRNA and miRNA expression data of 255 CRC patients were obtained from TCGA project [14]. The gene expression FPKM (fragment per kilobase per million mapped reads) values were calculated. The gene with a zero FPKM value in all samples was discarded. After normalization, 17,300 mRNAs and 612 miRNAs remained. To avoid infinite values, a value of 0.001 was added to the FPKM value before log 2 transformed for each gene.

### 2.2. miRNA-mRNA Association Analysis

The pairwise Pearson correlation coefficient between mRNA and miRNA expression was calculated based on 17,300 mRNAs and 612 miRNAs, yielding a correlation coefficient data matrix with 17,300 mRNAs in row and 612 miRNAs in columns. A Pearson correlation coefficient less than −0.5 was used as a cutoff to obtain the most probable biologically relevant miRNA-mRNA regulations. Data preprocessing and Pearson correlation coefficient calculation were performed in the R (https://www.r-project.org/) environment with its “base” function and “stat” packages. *χ*^2^ test was used to determine the correlation of miR-200c and clinical features.

### 2.3. Coexpression Gene Network Analysis

Gene coexpression networks were built according to the normalized gene expression values. We constructed the network adjacency between two genes, *i* and *j*, defined as a power of the Pearson correlation between the corresponding gene expression profiles according to the coexpression method [15]. By computing the correlation coefficient of these genes, we obtained the gene-gene coexpression adjacency matrix, *M*(*i*, *j*). Then, we only selected the strongest correlations (0.7 or greater) to be drawn in coexpression network graphs with CytoScape [16].

### 2.4. KEGG Pathway Enrichment Analysis of Coexpressed Genes

Coexpressed genes identified by correlation analysis were used to query the KEGG pathway database [17] to determine the biological function of these coexpressed genes. Enriched pathway was determined by both significant Fisher exact test (*p* value < 0.05), and at least 3 genes were involved in the pathway. The pathway enrichment analysis was performed by using “KEGG.db” and “KEGGprofile” packages in R project.

## 3. Results

### 3.1. Correlation Coefficient Revealed the Association between mRNA and miRNA

**Figure 1:**
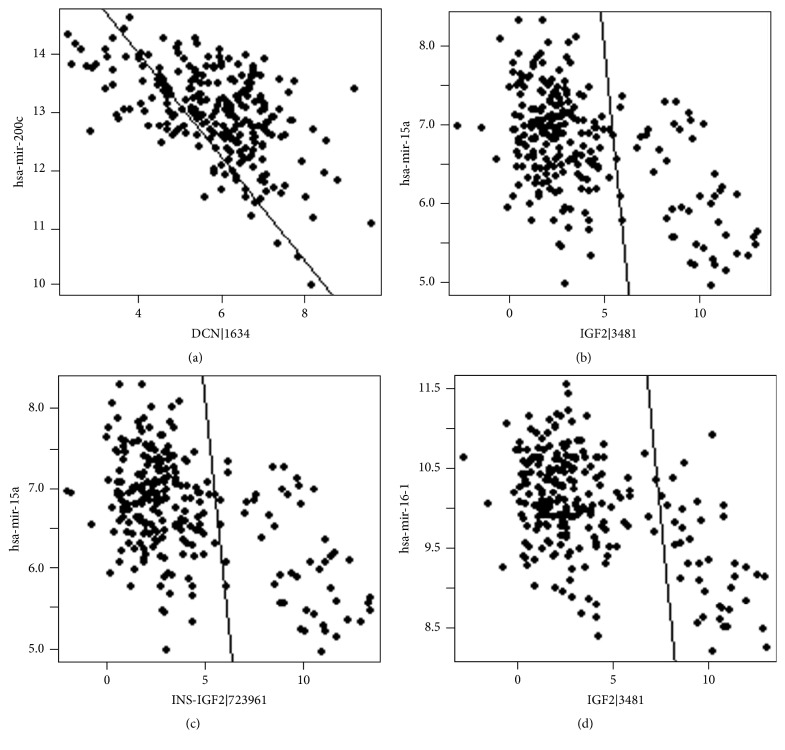
Scatter plot for miRNA and mRNA gene expression profiles. Cor indicates the Pearson correlation coefficient.

The TCGA colon cancer dataset included both mRNA and miRNA gene expression profiles of 255 tumor samples. Given the regulatory relationship between miRNA and mRNA, we assumed that the correlation between miRNA and the expression of its target genes was negative. By using a cutoff of negative Pearson correlation coefficient less than −0.5, 13 mRNA and miRNA association pairs were identified in colon cancer (Table 1), and the scatter plots of top four mRNA and miRNA pairs were shown in (Figure 1). The most significant association was identified between hsa-mir-200c and DCN (Decorin). Notably, previous studies also revealed that hsa-mir-200c was a very important regulator in colon cancer and significantly upregulated in tumor samples. However, DCN is a tumor suppressor gene in colorectal cancer [18]. In addition, the hsa-mir-200c was associated with the IGFBP7, PDLIM3, and SERPINF1 expression. Furthermore, the hsa-mir-15a was observed to correlate with the expression of IGF2. In the TCGA colon cancer study, the IGF2 was amplified and overexpressed.

### 3.2. The Association of miR-200c with Clinical Outcomes of CRC Patients

We obtained the clinical information from TCGA colon cancer dataset. The relevance of miR-200c expression level and patients' clinic pathological features was evaluated to reveal the possible influence of miR-200c on patients' clinical outcomes. The association of miR-200c with age, overall survival, tumor metastasis, colon polyps history, lymphatic invasion, tumor stages, and other clinical features was evaluated. *χ*^2^ test was used to determine the correlation (Table 2). Results indicated that colon polyps history was significantly (*p* < 0.05) related to the expression level of miR-200c. Furthermore, the overall survival (OS) was significantly correlated (*p* < 0.01).

### 3.3. Coexpression Networks in Colon Cancer

**Figure 2:**
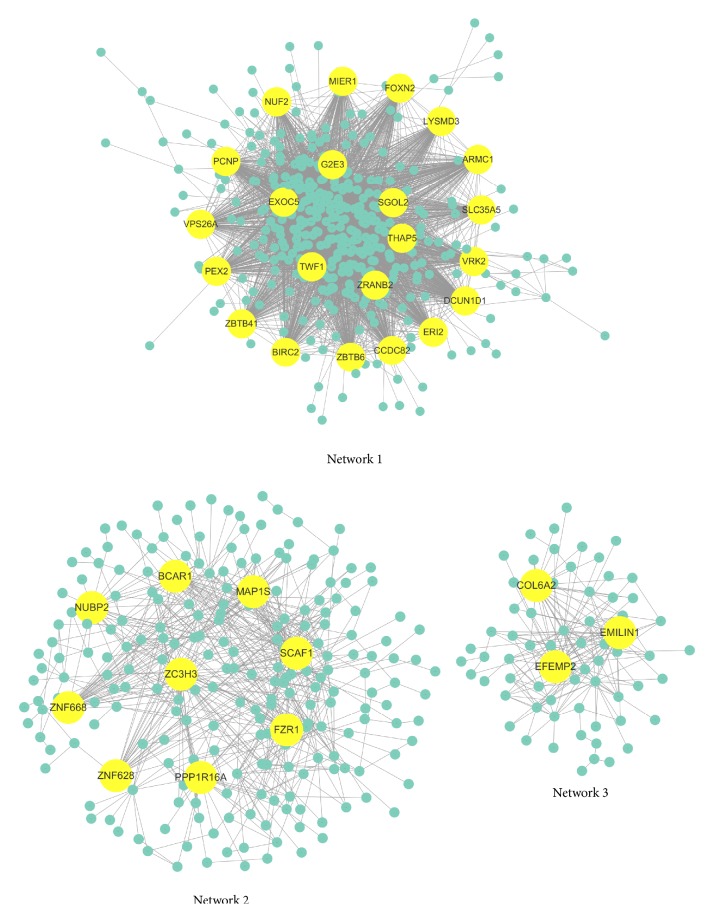
Coexpression network identified with mRNA expression of colon cancer. Node with interaction larger than 20 was colored in yellow.

In order to reveal the gene-gene interactions underlying in colon cancer pathogenesis, we constructed three coexpression networks according to the clustering of Pearson correlation of gene expression. Genes with similar function in a biological process were hypothesized to have similar expression patterns. Coexpression analysis identified gene-gene interaction network through the correlation of gene expression profile and clustering of thousands of transcript into a functional module. As shown in Figure 2, each node indicated a gene and two genes are connected by an edge according to the correlation coefficient (i.e., either positive or negative) which indicated the existence of interaction. The importance of a gene in the network was determined by the number of interactions associated with this gene. We identified 421 genes in coexpression network 1 and 318 genes involved in network 2. Within the network analysis, we focused on the genes that are linked with more than 20 neighbors. Here, we identified 22 hub genes in network 1, 9 hub genes in network 2, and 3 hub genes in network 3.

### 3.4. Pathway Enrichment Analysis for Coexpression Network

The three networks identified with gene expression of tumor tissues may contribute to the initiation and development of colon cancer. In order to characterize the molecular functions of the networks in colon cancer, the pathway enrichment analysis was performed. All the genes involved in the networks were used to query the KEGG database to identify enriched pathway. Significantly enriched KEGG pathways with Fisher exact *p* value were listed in Tables 3 and 4. The top enriched pathways of network 1 were primarily cell cycle and oocyte meiosis pathways, which indicated the cell proliferation related to colon progression. Among the enriched genes, CDK1 plays an important role in the cell cycle, while RB1 is a driver gene in several cancer types. In addition, CCNE2, PIK3CB, ITGAV, RB1, and BIRC2 involved in cancer pathway were also affected. For network 2 and network 3, endocytosis pathway was significantly enriched. Two cancer pathways, mTOR signaling pathway and NOTCH pathway, were affected as well. The function annotation analysis revealed the relationship between gene expression alteration of cell cycle and cancer pathways. The association between coexpressed genes and colon cancer biology indicated the networks were involved in molecular mechanism of colorectal cancer pathogenesis.

## 4. Discussion

Recent studies have shown the importance of miRNA and its regulatory network in colorectal carcinoma and the potential role of miRNA in colon cancer initiation and progression has been partly explored [13, 14, 19]. Here, we use both miRNA and mRNA expression data from TGCA project to reveal the gene regulatory and coexpression networks of colon cancer. In this study, we identified expression of 13 potential miRNAs and mRNA regulatory pairs, among which, notably, mir-200c and DCN have been previously reported in colon cancer. Three mRNA coexpression networks were constructed with mRNA gene expression profiles. In addition, the clinical relevance analysis demonstrated the association between colon polyps history and the expression of miR-200c. Our studies indicate the potential association between miR-200c, DCN, and CRC pathogenesis.

In this study, the gene expression of miRNA-200c was associated with DCN expression. The potential role of miR-200c has been well investigated in many cancer types, including colon cancer, ovarian cancer [20], lung cancer [21], gastric cancer [19, 22], and breast cancer [23]. The overexpression of miR-200c was observed in ovarian cancer and correlated with poor clinical outcome. It was reported as a driver of biological aggressiveness in ovarian cancer [24]. The miRNA-200c levels were also significantly correlated with patients' survival of gastric cancer [25]. MiR-200c functions as a driver gene in colon cancer and is associated with tumor apoptosis and metastasis [13]. However, an in vitro study demonstrated that miR-200c inhibits proliferative and invasive characteristic of colon cancer cell line [19]. It has also been reported that miR-200c is able to inhibit the metastasis of breast cancer cells [23] and inhibit their proliferation [23]. Some studies even drew conclusions that miR-200c could be a suppressor of tumor proliferation and invasion [25, 26]. Tissue specificity could be the cause of this paradoxical interpretation that miR-200c might play different roles in different cancers/organs of origin. Also, many of these experiments were performed in the cell lines, while cell line authentication information was not provided.

In addition, to date, miR-200 is divided into two groups according to a single nucleotide change in the seed sequence. MiR-141 and miR-200a are classified in group A, while miR-200b, miR-200c, and miR-429 are classified in group B. Notably, gene expression of miR-200a was also shown to be associated with 6 genes in this dataset. MiR-200a has been reported to be involved in the regulation of E-cadherin and involved in the pathogenesis of several types of cancer [27]. Previous study suggests that low miR-200a expression was associated with poor prognosis in CRC patients, and it was the regulator of epithelial–mesenchymal transition related gene expression [28]. Unlike miR-200c, the loss of miR-200a expression may contribute to the progression of breast cancer [29]. Moreover, the DCN (Decorin) gene encodes a member of the small leucine-rich proteoglycan family of proteins acting as a tumor suppressor gene lowly expressed in colon, breast, gastric, and ovarian cancer cells [24, 30–32]. It is reported to prevent metastatic spreading of breast cancer [33]. The overexpression of DNC in pancreatic cancer may play an important role in tumor suppressor effect towards the formation of pancreatic cancer cells [34]. In our analysis, the negative correlation between miR-200c and DCN was observed in CRC, which indicated that DCN could be a potential target of miR-200c. The experiment validation of this relationship will be performed in our further work.

Lastly, three coexpression networks have been constructed in this study. Hundreds of genes are involved in the networks, which may be related to the development and progression of colon cancer. Genes involved in cell cycle and oocyte meiosis pathways were significantly affected, which indicated that coexpression networks were related to tumor proliferation and growth. In addition, tumor pathways were also significantly enriched, including small cell lung cancer, NOTCH, and mTOR signaling pathways which were often altered in cancer tissues [35, 36]. The coexpression analysis could be an effective approach to identify gene network involved in cancer development.

Overall, we identified the miRNA and mRNA association in CRC, and the DCN might be a potential target of miR-200c, which indicated the important role of miR-200c and DCN in CRC. Clinical manifestations also implied the significant relation between colon polyps history, overall survival, and the expression level of miR-200c. This study provided an insight into cancer related mRNA coexpression network of CRC.

## Figures and Tables

**Table 1:** miRNA and mRNA correlation analysis.

	miRNA	Gene Symbol∣Entrez ID	Correlation coefficient
1	hsa-mir-200c	DCN∣1634	−0.54
2	hsa-mir-15a	IGF2∣3481	−0.51
3	hsa-mir-15a	INS-IGF2∣723961	−0.51
4	hsa-mir-200c	IGFBP7∣3490	−0.51
5	hsa-mir-200c	PDLIM3∣27295	−0.51
6	hsa-mir-200a	HSPB7∣27129	−0.51
7	hsa-mir-200c	SERPINF1∣5176	−0.50
8	hsa-mir-200a	SFRP4∣6424	−0.50
9	hsa-mir-200a	MAB21L2∣10586	−0.50
10	hsa-mir-552	TRIB2∣28951	−0.50
11	hsa-mir-625	C6orf15∣29113	−0.50
12	hsa-mir-200a	THBS4∣7060	−0.50
13	hsa-mir-16-1	IGF2∣3481	−0.50

**Table 2:** Clinical relevance of miR-200c.

	Number	miRNA expression		*p* value
		High	Low	
*Age*				**0.42**
>60	198	108	24	
≤60	31	90	27	
*Distant metastasis*				**1**
0	204	107	97	
1	41	22	19	
*Colon polyps history*				0.04^**∗**^
Yes	105	47	58	
No	133	78	55	
*Lymphatic invasion*				**0.1**
Yes	127	69	58	
No	101	43	58	
*Tumor stage*				**0.88**
I	50	24	26	
II	89	47	42	
III	65	36	29	
IV	41	22	19	
*Neoplasm cancer status*				**0.34**
With tumor	214	110	104	
Tumor free	32	13	19	
*Number of positive lymph nodes *				**0.34**
≥2	83	37	46	
<2	160	83	77	
*Vascular invasion present*				**0.35**
Yes	58	29	29	
No	151	63	88	
*Overall Survival*				**0.0025**
≤31 days (median)	156	66	90	
>31 days (median)	92	58	34	

*∗* indicates that colon polyps history was significantly (*p* < 0.05) related to the expression level of miR-200c.

**Table 3:** Pathway enrichment analysis of network 1.

Term	Count	%	*p* value	Genes
hsa04110: Cell cycle	8	1.91	4.62*E* − 03	CCNE2, CDK1, E2F5, DBF4, TTK, ANAPC10, RB1, CDC27
hsa04114: Oocyte meiosis	6	1.43	3.61*E* − 02	CCNE2, CDK1, SLK, FBXO5, ANAPC10, CDC27
hsa00230: Purine metabolism	7	1.67	4.19*E* − 02	POLR3G, POLE2, POLR2K, NT5C3, PDE4D, RRM2B, PPAT
hsa05222: Small cell lung cancer	5	1.19	5.12*E* − 02	CCNE2, PIK3CB, ITGAV, RB1, BIRC2
hsa00240: Pyrimidine metabolism	5	1.19	7.37*E* − 02	POLR3G, POLE2, POLR2K, NT5C3, RRM2B
hsa04120: Ubiquitin mediated proteolysis	6	1.43	7.82*E* − 02	TRIM37, UBE2W, UBA6, ANAPC10, BIRC2, CDC27
hsa00512: O-Glycan biosynthesis	3	0.72	8.89*E* − 02	GALNT3, GALNT7, C1GALT1
hsa05200: Pathways in cancer	10	2.39	9.53*E* − 02	CCNE2, NRAS, HIF1A, PIK3CB, ITGAV, BRCA2, KITLG, RB1, BIRC2, FZD6

**Table 4:** Pathway enrichment analysis of network 2 and network 3.

Term	Count	%	*p* value	Genes
hsa04144: Endocytosis	9	2.85	9.79*E* − 03	ARFGAP1, GIT1, HRAS, AP2A1, GRK6, RAB11B, HGS, EPN1, SH3GL1
hsa04150: mTOR signaling pathway	5	1.58	9.86*E* − 03	ULK1, STK11, TSC2, MLST8, RPTOR
hsa04330: NOTCH signaling pathway	4	1.27	4.06*E* − 02	NOTCH3, RFNG, NCOR2, DVL1

## Abbreviations

CRC: Colorectal carcinoma
DCN: Decorin
CEA: Carcinoembryonic antigen
PSA: Prostate-specific antigen
CA: Carcinoma antigens
CtDNA: Circulating tumor DNA
CTCS: Circulating tumor cells
CLL: Chronic lymphocytic leukemia
PDOX: Patient-derived orthotopic xenograft.
